# Quantitative intratumoural microdistribution and kinetics of ^131^I-huA33 antibody in patients with colorectal carcinoma

**DOI:** 10.1186/s13550-014-0022-x

**Published:** 2014-05-30

**Authors:** Marika Ciprotti, Geoffrey Chong, Hui K Gan, Anthony Chan, Carmel Murone, Duncan MacGregor, Fook-Thean Lee, Terrance G Johns, Joan K Heath, Matthias Ernst, Antony W Burgess, Andrew M Scott

**Affiliations:** 1 Ludwig Institute for Cancer Research, Austin Health, Melbourne 3084, Australia; 2 Department of Anatomical Pathology, Austin Health, Melbourne 3084, Australia; 3 Monash Institute for Medical Research, Melbourne 3168, Australia; 4 Walter and Eliza Hall Institute of Medical Research, Melbourne 3052, Australia; 5 Department of Nuclear Medicine and Centre for PET, Austin Health, Melbourne 3084, Australia

**Keywords:** Monoclonal antibody, Immunotherapy, Colon cancer, Microdistribution

## Abstract

**Background:**

The ability of recombinant antibodies to adequately penetrate into tumours is a key factor in achieving therapeutic effect; however, the behaviour of antibodies at a cellular level in tumours is poorly understood. The purpose of this study was to investigate those factors that influence the macroscopic and microscopic intratumoural distribution of an IgG1-humanized antibody, huA33, in colorectal tumours.

**Methods:**

Twelve patients were infused with radiolabelled huA33 at 7 days prior to elective surgery for colorectal carcinoma. Macroscopic huA33 uptake was determined by both gamma well counter and autoradiography measurements of the resected tumour specimens. Microscopic uptake was then quantitated at a cellular level and compared to vascular penetrance. The impact of variation in tumour antigen (GPA33) expression, tumour size, specimen type (primary vs metastatic), presence of macroscopic necrosis, and tumour vasculature on huA33 uptake were examined.

**Results:**

The I-huA33 uptake in whole tumour sections was (mean ± SD) 5.13 ± 2.71 × 10^−3^% injected dose per gram (ID/g). GPA33 was expressed in all viable tumour cells, and huA33 uptake was excellent regardless of tumour size and specimen type. In tumours with macroscopically evident central necrosis (*n* = 5), huA33 uptake in tumour necrotic centres was lower than in viable peripheries (0.606 ± 0.493 vs 2.98 ± 2.17 × 10^−3^%ID, *p* = 0.06). However, when corrected for low cell viability in necrotic centres, uptake of huA33 at the cellular level was highly comparable to that in the more viable tumour periphery (7.10 ± 5.10 × 10^−9^ vs 3.82 ± 3.67 × 10^−9^%ID/cell, *p* = 0.4). In the five patients who exhibited macroscopic necrosis in their tumours, huA33 showed excellent tissue penetration, with a maximum penetration distance of 26 μm in peripheral tumour regions and 118 μm in central regions. No correlation was observed between ^131^I-huA33 uptake in tumour on a cellular basis and tumour vascularity.

**Conclusions:**

In patients with colorectal carcinoma, monoclonal antibody huA33 effectively targets viable tumour cells in all cellular milieus examined, including effective penetration into necrotic tumour centres, a novel and therapeutically important finding.

## Background

There is increasing interest in the use of humanized monoclonal antibodies to treat epithelial cancers. This technique is attractive owing to limited chemotherapy options for many such cancers and the relatively low toxicity of using tumour-specific radiolabeled monoclonal antibodies. Many unlabeled monoclonal antibodies (mAbs) do not demonstrate clinically significant anti-cancer activity, despite induction of complement-dependent cytotoxicity (CDC) and antibody-dependent cellular cytotoxicity (ADCC). Hence, attempts have been made to utilize the targeting ability of mAbs to deliver radioactive isotopes or other cytotoxic molecules to individual cancer cells [1,2].

In order for antibody therapy approaches to be effective, it is necessary for the antibody to adequately penetrate within tumours after intravenous administration. Previous studies have found variable uptake in epithelial tumours depending on tumour size, histological type, vascularity, degree of necrosis, antigen expression, antibody size and affinity, antibody internalization, and other factors that are less well understood [3-10]. Suboptimal response to antibody therapy may be due to poor or non-uniform penetration of antibody into certain tumour areas such as central necrotic regions which lack an adequate blood supply [11]. Effective intratumoural penetration is even more important for antibody-drug conjugates which rely on direct cellular contact to effect cell kill. Despite this, detailed understanding of the impact of antigen expression and vascular density on the distribution of humanized antibodies in tumours, and penetrance into metastatic lesions following systemic infusion, is lacking.

One of the most promising targets in colorectal cancer is the A33 antigen (hereafter referred to as GPA33): a transmembrane glycoprotein of the immunoglobulin superfamily with a molecular weight of 43 kDa [12-14]. GPA33 consists of two extracellular Ig domains, a single transmembrane domain, and a short intracellular tail containing four acylation sites proximal to the transmembrane domain [12,14]. Extensive immunohistochemical analysis of malignant and normal tissues has demonstrated that the antigen is homogeneously expressed by more than 95% of colon cancers and in the normal intestinal mucosa, but not in other epithelial tissues [15,16]. Previous radioimmunotherapy studies using iodinated murine A33 mAb have shown the therapeutic potential of the GPA33 antibody system in patients with metastatic colorectal cancer [17-19].

HuA33 is a humanized monoclonal antibody (mAb) directed against GPA33 and has been studied in phase I trials in patients with colorectal cancer [20-25]. Biodistribution studies with ^131^I and ^124^I have shown selective and rapid localization of huA33 to colorectal carcinoma on imaging [20,22,23,26]. Tumour localization of huA33 was observed within 24 to 48 h post injection, increased over 7 days, and was retained in tumour for over a month following infusion [20,22,23]. As expected, the uptake in the colon and small bowel was also seen, but decreased over 7 to 10 days. Importantly, there was no evidence of non-selective binding to normal tissues. The present study examined tumours from one of these trials [23] in order to explore the intratumoural microdistribution and kinetics of huA33 *in vivo*, with particular focus on the quantitative cellular uptake and vascular penetrance of huA33 in viable and necrotic tumours.

## Methods

### Patient eligibility and treatment

Patient eligibility and treatment has previously been described [23]. In brief, patients with colorectal cancer who were also scheduled for resection of primary or secondary tumour, or for intrahepatic artery catheter insertion, were recruited. Patients were required to be between 18 and 70 years old. Any chemotherapy, radiotherapy, and immunotherapy had to be completed at least 4 weeks prior to trial entry. Patients had to have a Karnofsky performance status of ≥70% and acceptable major organ function: serum creatinine <0.2 mmol/L, serum bilirubin <20 mmol/L, granulocytes >1.5 × 10^9^/L, platelets > 100 × 10^12^/L, prothrombin time <1.3× control. All patients gave written informed consent to participate in this study. The protocol was approved by the Human Research Ethics Committee of the Austin Hospital, Melbourne, Australia and the Protocol Review Committee, Ludwig Cancer Research, New York, USA.

Eligible patients received a single intravenous infusion of 296 to 370 MBq (8 to 10 mCi ) of ^131^I^−^ and 37 to 74 MBq (1 to 2 mCi) of ^125^I^−^ conjugated to huA33 (IgG1) over a 30-min period, at antibody dose levels of 0.25, 1, 5, and 10 mg/m^2^. ^125^I^−^ and ^131^I^−^ were used to assist with both imaging and autoradiography studies. Seven days later, surgery was performed and tumour samples were obtained for histology and autoradiography. Surgery comprised complete tumour resection (of either the primary tumour or metastases) or biopsy of accessible tissue for patients having surgery only for placement of intrahepatic artery catheter.

All patients underwent gamma camera imaging on day 0 after infusion of ^131^I-huA33 and on at least three further occasions up to day 7 following infusion. Single photon emission computed tomography (SPECT) images of a region of the body with known tumour were also obtained on at least one occasion during this period. All gamma camera images were acquired on a dual-headed gamma camera (Trionix Research Laboratories, Twinsburg, OH, USA). Whole body images were performed as sweeps in a 1,024 × 256 bit matrix, and a standard of known ^131^I activity was included in the field of view to allow dosimetry calculations. In selected patients, resected tumours were also imaged on a gamma camera to aid in determination of regional localisation of ^131^I-huA33.

### Quantification of macroscopic ^131^I-huA33 tumour uptake

The magnitude of ^131^I-huA33 uptake in whole tumour was assessed using gamma well counter measurements (Packard Instruments, Melbourne, Australia) in all 12 patients. Each tumour was divided into subsections which were weighed individually and counted in a gamma counter (set to the ^131^I window) together with the appropriate counting standards. Results were expressed as percent injected dose per gram (%ID/g) tissue.

The magnitude of ^131^I-huA33 uptake was confirmed by whole tumour autoradiography in four patients whose tumours were suitable for evaluation using this technique (patients 4, 5, 7, 11); two other patients (patients 1 and 2) could not be assessed due to technical problems and six patients (patients 3, 6, 8 to 10, 12) had tumours which were too large to allow whole tumour radiography. For autoradiography, the resected tissue was weighed and bisected with a portion fixed in 10% neutral buffered formalin, embedded in paraffin, and stored at room temperature. Tissue sections were cut at a thickness of 5 μm and mounted onto SuperFrost® Plus slides (Menzel-Glaser, Braunschweig, Germany). Paraffin sections were exposed to x-ray film (Agfa Mammoray, Mortsel, Belgium or Hyperfilm-βmax, Amersham International plc, Aylesbury, UK) for 4 to 21 days. X-ray film was then developed in D19 developer (Eastman Kodak Co., Rochester, NY, USA) and fixed in Ilford Rapidfix (Ilford Imaging Australia, Mt. Waverley, Victoria, Australia). A set of radioactivity standards was prepared by applying known amounts of ^125^I/^131^I to discs of tissue sections 3 mm in diameter and 5-μm thick mounted onto SuperFrost® Plus slides. These standards were exposed simultaneously with the radioactive biopsy sections to allow quantitation of antigen density by computerized densitometry using an image analysis system (VideoPro32, Leading Edge, Adelaide, Australia). The grey value of the pixels on x-ray film was converted to %ID/g for quantitation of antibody uptake. High-power images of haematoxylin and eosin (H&E) sections were captured using a microscope and attached digital camera (Olympus, Tokyo, Japan).

### Assessment of GPA33 expression

The GPA33 expression on cells was probed using murine anti-A33 antibody (Ludwig Cancer Research, New York, NY, USA) in fresh frozen sections. The concentrations used were optimized for GPA33 detection (2.5, 5, and 10 μg/ml for 30 min) and bound antibody detected by application of a biotinylated sheep-anti-mouse IgG (1/500 dilution, Southern Biotechnology Associates Inc. Birmingham, AL, USA) for 30 min followed by streptavidin peroxidase complex (1/1,000 dilution, Silenus, Amrad Operations P/L, Melbourne, Australia). Negative controls for both antibodies were either secondary antibody alone or isotype control antibodies.

### Assessment of microvessel density and intercapillary distance for CD31-positive vessels

CD31-positive blood vessels were determined by staining for CD31 expression (Clone JC70A; Dako A/S Glostrup, Denmark) in paraffin sections which were deparaffinized and rehydrated by microwaving in a citrate buffer (pH 6.0) for 10 min. Sections were then treated with 3% H_2_O_2_ for 10 min to eliminate endogenous peroxidase activity, used 0.1% avidin and 0.01% biotin for 10 min to block endogenous biotin and avidin activity respectively, and treated with protein blocking agent (Immunon™, Shandon, Pittsburgh, PA, USA) at a 1:1 dilution for 10 min to reduce non-specific binding. JC70A was used at 3.5 μg/ml for 18 h at room temperature, and bound antibody was detected using the LSAB®2 Peroxidase System (Dako, Denmark). Staining was then visualized using chromogen 3-amino-9-ethylcarbazole (AEC; 0.4%, Sigma Chemical Co., St. Louis, MA, USA) for 20 min and counterstained with Mayer’s haematoxylin. Digital images of the sections were captured using an Aperio Scanscope (Leica Biosystems, Wetzlar, Germany) and the images were viewed using the Aperio ImageScope software (Aperio Technologies, Vista, CA, USA). Using these images, microvessel density (MVD) was determined by manually counting CD31-positive vessels in five ×0.118 mm^2^ randomly selected fields at ×200 magnification and results expressed as mean number of vessels per square millimetres. Then, intercapillary distance (ICD) was determined using the Aperio ImageScope ruler tool and by measuring the distance between a randomly selected index vessel and the four nearest vessels in each quadrant of a randomly selected field, with results expressed as the mean of these distances.

### Quantification of microscopic ^131^I-huA33 tumour uptake and intratumoural penetration

A detailed analysis was undertaken in patients (3 to 6, 12) whose tumours contained macroscopic areas of necrosis by comparing five representative fields in viable peripheral tumour tissue with five fields in central necrotic tissue. Mean percent injected dose per cell was determined by dividing the huA33 uptake (%ID/g), calculated using the autoradiographic images, by the total number of cells in each field, determined by manual cell counting in digitized high-power H&E images. Subsequently, images of sections stained for CD31-positive blood vessels were superimposed on their autoradiographic images and five representative sections from the viable peripheral tissues were compared with a similar number of sections from the necrotic central tissue. The mean antibody penetration was determined using Aperio ImageScope Ruler Tool and measuring the distance between five random areas of huA33 uptake as detected by autoradiography and the nearest CD31-positive blood vessel as determined by the superimposed image.

### Statistical analysis

All statistical analyses were performed with the statistical software IBM® SPSS® Statistics version 18.0 (SPSS Inc., Chicago, IL, USA). All groups were verified for normal distribution before statistic calculations. All comparisons across groups were determined using a non-parametric Mann-Whitney *U* test. All statistical tests were conducted using a two-sided alpha level of 0.05.

## Results

**Figure 1:**
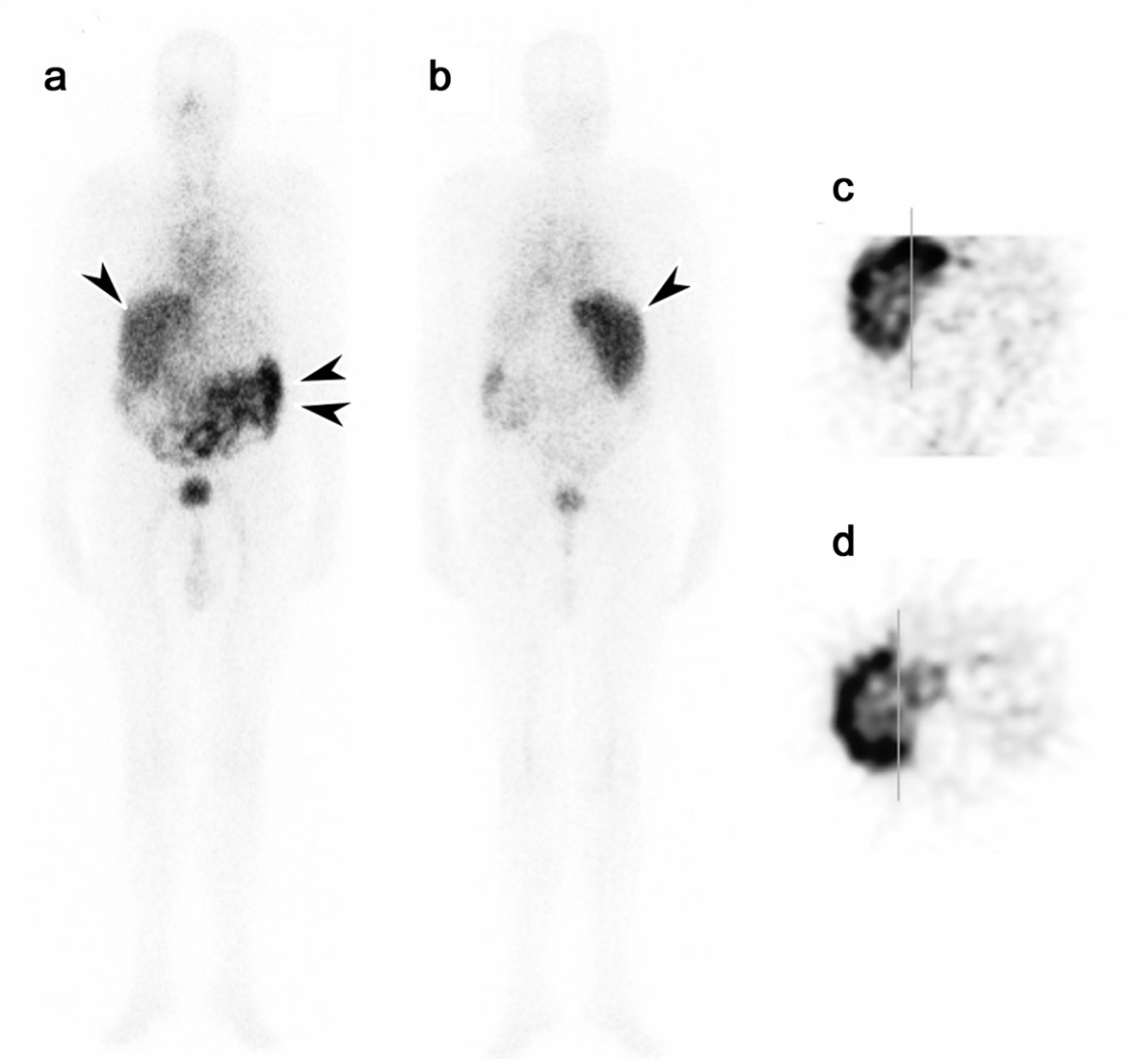
**Gamma camera and SPECT imaging after infusion of**^**131**^**I-huA33.** Whole body gamma camera image **(a)** anterior and **(b)** posterior of patient 6 at 6 days following infusion of ^131^I-huA33. Excellent uptake of ^131^I-huA33 in extensive liver metastases involving the entire right lobe of liver (arrow) is evident, with some bowel uptake (double arrows) also seen. Coronal SPECT **(c)** and transverse SPECT **(d)** through the right lobe of liver showing ^131^I-huA33 uptake in metastatic disease throughout the right lobe of liver, with some central reduced uptake due to necrosis (confirmed on histology of resected hepatic lobe).

**Figure 2:**
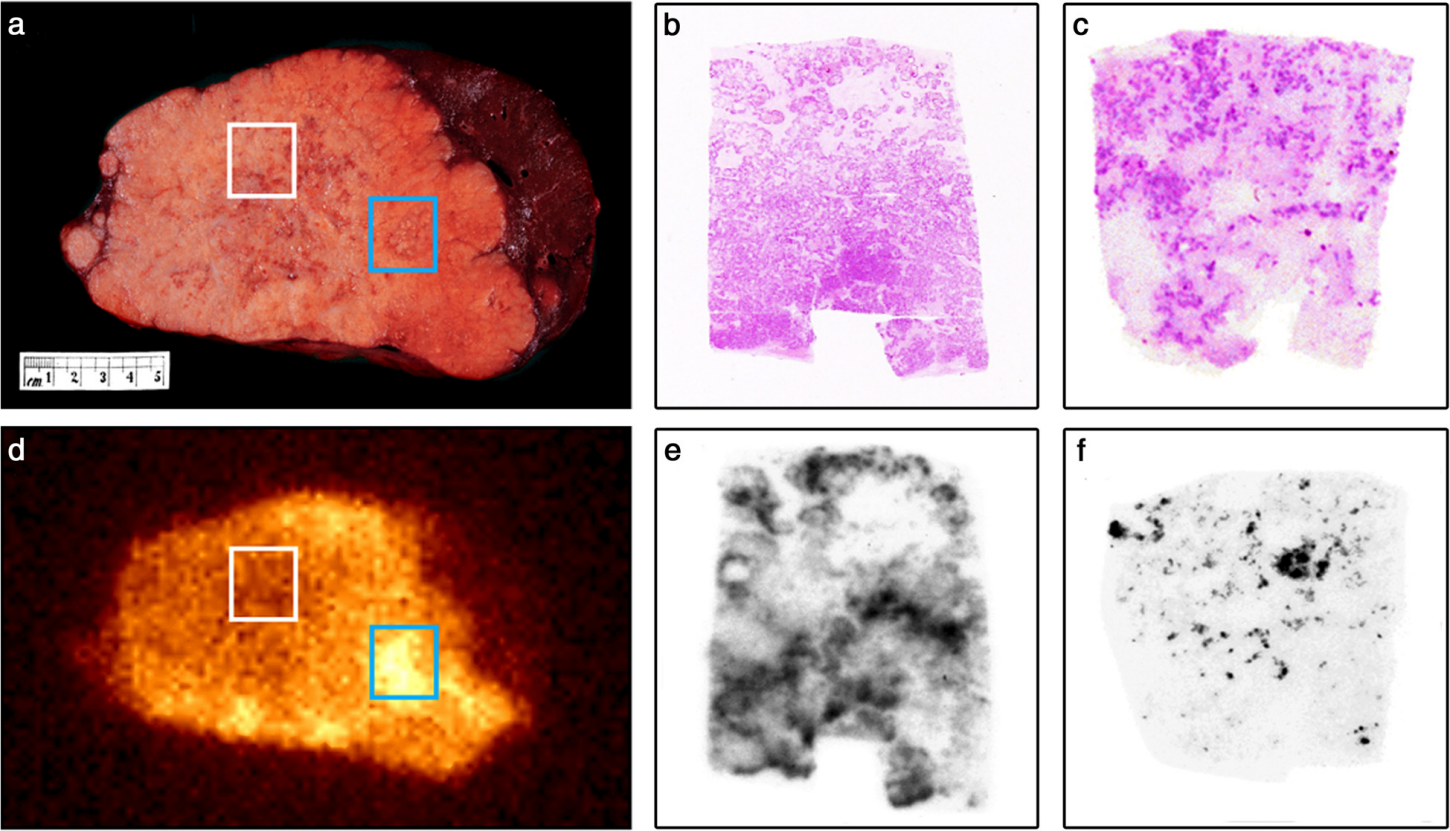
**Representative image of tumour with macroscopic areas of necrosis.** Tumour analysis from patient 6. **(a)** Macroscopic view of *ex vivo* liver tissue with extensive tumour infiltration, including viable (blue box) and necrotic (white box) areas. **(b)** H&E section (magnification ×2) of tissue from areas of strong ^131^I-huA33 uptake in macroscopically viable tumour [blue box in **(a)** and **(d)**] showing numerous viable tumour clusters with occasional necrotic/acellular areas. **(c)** H&E section (magnification ×2) of tissue from area of reduced uptake of ^131^I-huA33 [white box in **(a)** and **(d)**] showing areas of extensive stroma and necrosis but with some islands of viable tumour. **(d)** Corresponding *ex vivo* gamma camera image of tumour from **(a)** showing ^131^I-huA33 uptake in all macroscopic tumour but highest uptake in viable areas (blue box), lesser uptake in necrotic areas (white box), and no specific uptake in normal liver tissue (upper right edge of specimen). **(e, f)** Corresponding autoradiographs from **(b)** and **(c)**, respectively, showing high uptake in all viable tumour islands, demonstrating that the apparent lack of uptake in macroscopically necrotic tissue is a function of reduced cell number rather than reduced antibody penetration or cellular uptake.

**Figure 3:**
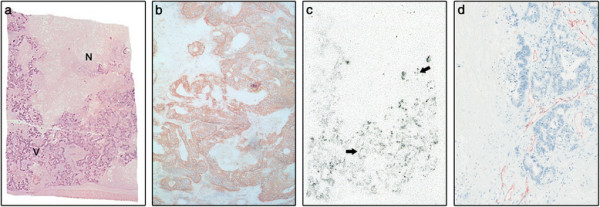
**Representative tumour biopsy (patient 12). (a)** Macroscopic view of *ex vivo* liver tissue with tumour infiltration, including viable (V) and necrotic (N) areas. **(b)** Strong and homogenous GPA33 expression in viable tumour, demonstrated by IHC. **(c)** Autoradiograph of corresponding tissue in **(a)** showing high uptake of ^131^I-huA33 in viable areas and similar degree of uptake by viable cell clusters of tumour within morphologically necrotic areas of the tumour (arrows). **(d)** IHC section showing CD-31 positive vessels predominantly in viable areas of tumour.

The demographics and tumour characteristics of the 12 patients in this protocol are shown in Table 1. Gamma camera and SPECT imaging after infusion of ^131^I-huA33 was sensitive and specific for tumour (representative images are shown in Figure 1). All lesions in 12 patients that were detected by imaging were confirmed to be of primary or metastatic colorectal cancers at surgery. Patient 7 had a total colectomy yielding three small primary adenocarcinomas. Tumours from seven patients (nos 1, 2, 3, 4, 5, 6, and 12) were noted by the pathologist to have central areas of necrosis, with the tumour from patient 6 also having stromal elements admixed with necrotic tissue. A representative image of a tumour with macroscopic areas of necrosis is shown in Figure 2. Importantly, all specimens showed that viable tumour cells expressed GPA33 as determined by immunohistochemistry (IHC) (Figure 3).

**Table 1:** Patient and tumour characteristics according to assigned huA33 dose level

**Patient no.**	**huA33 antibody dose level (mg/m**^**2**^**)**	**Sex**	**Age (years)**	**Location of tumour resection or biopsy**	**Reason for operation**	**Tumour volume (cm**^**3**^**)**^**a**^
1	0.25	M	63	Liver	Bx	270
2	0.25	F	68	Lung	M	37
3	0.25	M	56	Liver	Bx	48
4	1	M	61	Liver	Bx	132
5	1	F	52	Liver	M	26
6	1	M	66	Liver	M	2,099
7	5	M	62	Colon	P	4
8	5	F	48	Liver	Bx	1,770
9	5	F	60	Liver	Bx	135
10	10	F	39	Liver	M	80
11	10	M	59	Liver	Bx	39
12	10	F	49	Liver	Bx	131

^a^Total volume of tumour identified and measured on CT scan, or at pathology (Pt. 7). Bx, biopsy at time of intrahepatic artery port insertion; M, metastectomy; P, surgery for primary colon cancer.

Table 2 shows ^131^I-huA33 uptake in tumour measured by radioactivity gamma well counter and autoradiography. The mean whole tumour ^131^I-huA33 uptake measured by gamma well counter was 5.13 ± 1.76 × 10^−3^%ID/g, and mean whole tumour ^131^I-huA33 uptake measured by autoradiography was virtually identical at 5.12 ± 2.71 × 10^−3^%ID/g. Intratumoural uptake of ^131^I-huA33 was compared in the periphery and centre of these tumours. There was reduced uptake in tumour centres compared to tumour periphery (1.61 ± 1.76 × 10^−3^%ID/g vs 4.11 ± 2.53 × 10^−3^%ID/g, *p* = 0.07). As a subset of patients exhibited macroscopic necrosis in their tumours, these results were stratified by the presence of tumour necrosis (Table 2). This stratification revealed that patients whose tumours were macroscopically viable in both the centre and the periphery had minimal difference in uptake between regions (4.12 ± 0.212 × 10^−3^%ID/g vs 5.39 ± 2.63 × 10^−3^%ID/g, *p* value not available due to limited patient numbers), whereas patients whose tumours had necrotic centres showed a trend towards less uptake in the tumour centres than the tumour periphery, though this was not significantly different (0.606 ± 0.493 × 10^−3^%ID/g vs 2.98 ± 2.17 × 10^−3^%ID/g, *p* = 0.06). However, the number of viable tumour cells was also found to be significantly lower in central necrotic areas compared to peripheral viable areas (Table 3; 54 ± 42 vs 313 ± 146, *p* < 0.001). Given the significantly different numbers of viable cells in necrotic versus viable regions, huA33 uptake was corrected for the differing cell count by calculating the percent injected dose per cell (%ID/cell). Interestingly, cellular uptake of huA33 in tumour central necrotic areas was not significantly different from that in peripheral viable areas (7.10 ± 5.10 *×* 10^−9^ vs 3.82 ± 3.67 × 10^−9^%ID/cell respectively, *p* = 0.4).

**Table 2:** **Percent injected**^**131**^**I-huA33 dose per gram of tumour measured by gamma well counter and autoradiography**

**Patient no.**	**Range of tumour uptake %ID/g × 10**^**−3**^**(gamma well counter)**	**Whole tumour %ID/g × 10**^**−3**^**(autoradiography)**	**Tumour centre %ID/g × 10**^**−3**^**(autoradiography)**	**Tumour periphery %ID/g × 10**^**−3**^**(autoradiography)**	***p*** **value**^**d**^
1	NE^a^	NE^a^	NE^a^	NE^a^	
2	NE^a^	NE^a^	NE^a^	NE^a^	
3	2.7-5.3	NE^c^	0.556 (N)	0.683	
4	5.3-14.1	3.09	0.947 (N)	3.24	
5	3.2-7.6	0.837	0.180 (N)	1.02	
6	1.7-5.0	NE^c^	1.25 (N)	4.06	
7a^b^	5.2-6.5	5.77	NE^c^	NE^c^	
7b^b^	NE^c^	6.22	NE^c^	NE^c^	
7c^b^	5.2-6.5	6.37	NE^c^	NE^c^	
8	1.4-4.5	NE^c^	4.27 (V)	3.53	
9	3.2-5.5	NE^c^	3.97 (V)	7.25	
10	0.6-5.1	NE^c^	NE^c^	7.20	
11	9.5-12.4	8.43	NE^c^	NE^c^	
12	3.6-8.1	NE^c^	0.0970 (N)	5.90	
Mean ± SD (N)			0.606 ± 0.493	2.98 ± 2.17	0.06
Mean ± SD (V)			4.12 ± 0.212	5.39 ± 2.63	NA^e^
Mean ± SD (all)	5.13 ± 1.76	5.12 ± 2.71	1.61 ± 1.76	4.11 ± 2.53	0.07

^a^Data from patients 1 and 2 were not available due to technical problems; ^b^patient 7 had three primary colonic tumours resected and were analyzed separately; ^c^the tumours from patients 3, 6, 8, 9, 10, and 12 were too large for whole tumour autoradiographs to be constructed; tumours from patient 7 and 11 were too small to determine central and peripheral areas; there was no tissue from patient 10 labelled as ‘tumour centre’; ^d^*p* value comparing 131I-huA33 uptake in tumour centres and tumour peripheries using Mann-Whitney *U* test; ^e^*p* value not available due to limited patient numbers. %ID/g, percent injected dose per gram; N, necrotic areas; V, viable areas; NE, not evaluable; SD, standard deviation; NA, not applicable.

**Table 3:** Analysis of tumour cell density, vascularity, and antibody penetrance

**TUMOUR CELL DENSITY**			
**Patient no.**	**Mean number of viable tumour cells/field of viable peripheral tumour areas**^**a**^	**Mean number of viable tumour cells/field of necrotic central tumour areas**^**a**^	**p-value**^**b**^
3	388±185	32±33	
4	408±170	80±33	
5	249±128	28±33	
6	243±34	54±17	
12	274±127	79±62	
Mean±SD	313±146	54±42	P<0.001
**MICROVESSEL DENSITY(MVD)**			
**Patient no.**	**Mean number of vessels/mm**^**2**^** in peripheral tumour areas**	**Mean number of vessels/mm**^**2**^**in central tumour areas**	**p-value**^**b**^
3	54±23	21±16	
4	52±12	13±6.4	
5	64±27	20±9	
6	39±6	17±15	
12	52±23	23±8	
Mean±SD	52±20	19±11	P<0.001
**ANTIBODY PENETRATION DISTANCE**			
**Patient no.**	**Mean distance cell-vessel (μm) in peripheral tumour areas (range)**	**Mean distance cell-vessel (μm) in central tumour areas (range)**	**p-value**^**b**^
3	5.5±3 (2.5-10.5)	48.7 ±23.8 (23.8-79.4)	
4	6.7±2.2 (3.9-10)	82.5±29.3 (45.1-118)	
5	14.7±7.8 (3.3-25.2)	53.9±17.4 (33.6-80)	
6	13.7±4.2 (9-18.4)	46.8±8.2 (39.1-59)	
12	11.7±9 (1.7-26)	41.1±14.2 (20.3-57.2)	
Mean±SD	10.5±6.6	54.6±23.5	P<0.001
**INTERCAPILLARY DISTANCE (ICD)**			
**Patient no.**	**Mean ICD (μm) in peripheral tumour areas**	**Mean ICD (μm) in central tumour areas**	**p-value**^**b**^
3	52.6±22.1	205.6±11.1	
4	63.2±33.5	410.2±135.5	
5	43.3±15.5	91.7±25.1	
6	103.4±37.8	185±58.4	
12	82.9±33.6	195.1±112.2	
Mean±SD	69.4±34.5	207.4±121.7	P<0.001

^a^Field area = 0.118 mm^2^.

^b^P-value comparing central versus peripheral tumour areas using Mann-Whitney U Test.

We next examined whether there were differences in MVD between these tumours (Table 3). There was a corresponding reduction in MVD in necrotic versus viable tumour (19 ± 11 vs 52 ± 20 vessels/mm^2^, *p* < 0.001). Interestingly, we observed that MVD in stromal tissue was approximately threefold higher than in adjacent peripheral viable tumour. The mean ICD was also significantly wider in the necrotic tumour than areas of viable tumour, being 207.4 ± 121.7 μm and 69.4 ± 34.5 μm, respectively (*p* < 0.001).

Given that that huA33 uptake was preserved despite the relative hypovascularity of the central necrotic regions, we examined whether antibody penetration was different in central necrotic versus peripheral viable tumour regions. We found that huA33 was able to penetrate further in necrotic areas rather than viable areas (54.6 ± 23.5 vs 10.5 ± 6.6 μm respectively, *p* < 0.001). The greatest absolute distance the antibody penetrated was 118 μm in the centre of necrotic tumours and 26 μm in the periphery.

## Discussion

This study clearly demonstrates the ability of an IgG1 antibody, huA33, to rapidly penetrate at high concentration the central necrotic areas of large metastatic colorectal tumours. At a cellular level, huA33 can target viable tumour clusters at a similar concentration in both necrotic and viable tumour areas. Importantly, huA33 can traverse large distances from intratumoural blood vessels, independent of the interstitial pressure effects present in central necrotic regions of tumours. These novel observations highlight the ability of a high affinity and low internalizing intact antibody to effectively distribute throughout large tumours and target viable tumour cells at relatively uniform and high concentration.

Initial histologic and quantitative autoradiography analysis of huA33 showed that uptake in necrotic areas was moderate (0.606 ± 0.493 × 10^−3^%ID/g), suggesting that intratumoural interstitial pressure may reduce antibody penetration. However, detailed histologic and IHC analysis demonstrated that necrotic tumour centres had significantly lower number of viable tumour cells compared to viable peripheral regions and when corrected for the number of viable tumour cells, uptake of huA33 in necrotic areas was comparable to that in peripheral viable tumour areas (7.10 ± 5.10 *×* 10^−9^ vs 3.82 ± 3.67 × 10^−9^%ID/cell respectively, *p* = 0.4). Whilst most published studies have shown reduced antibody uptake in necrotic areas, Boxer et al. found that, in some cases, patients infused with antibody to CEA had increased concentrations of antibody in necrotic more than viable areas of tumour [4]. However, they did not correct for cell density. In contrast, we found that tumours without central necrosis demonstrated relatively even antibody distribution following intravenous administration of the radiolabelled antibody. Areas of necrosis had lower absolute antibody uptake, but upon examining uptake per viable tumour cell, the uptake was similar to homogeneous areas of viable tumour cells.

Interestingly, the ability of huA33 to penetrate into the necrotic centres of large tumours and localize to viable tumour cells was not reduced despite the demonstrably poorer perfusion of these necrotic central areas (long ICD and low MVD) compared to the periphery (short ICD and high MVD). Other studies have not found consistent relationships between degree of vascularity and antibody uptake in tumours [3-5,8,9,27]. Given the avascular nature of the necrotic tumour centre, we conclude that huA33 can penetrate long distances from blood vessels before binding to tumour cells. HuA33 exhibited an average maximum penetration distance of 82.5 ± 29.3 μm, with upper limit of this range being 118 μm. This distance might be even larger if the nearest blood vessels in the necrotic centre have impaired function and the antibodies therefore may diffuse in from the vascular capsule of the tumour. Given that huA33 is a high-affinity antibody [21], this long penetration distance is an unexpected finding. This is because very high affinity interactions between antibodies and tumour antigens are predicted to impair efficient tumour penetration of the monoclonal antibodies and thus diminish effective *in vivo* targeting [28-32]. Similar findings were recently reported by Rudnick et al. [27], who studied the impact of affinity on the *in vivo* tumour-targeting properties of anti-HER2 antibodies. They found that the highest affinity mAbs exhibited an average penetration of 20.4 ± 7.5 μm from tumour blood vessels. Conversely, lowest affinity mAbs revealed the greatest average penetration distance (84.8 ± 12.8 μm) and the mAb with a moderate monovalent affinity penetrated to an average distance of 59.7 μm. It is therefore likely that the low internalization rate of huA33 [33] contributes to its penetrance capability in tumour, as internalization plays an important role in antibody capture and degradation [27].

Other intratumoural factors may contribute to the long penetration distance of huA33, such as an enhanced permeability and retention (EPR) effect which is known to lead to a prolonged retention of drugs into tumour interstitium [34,35]. Whilst the EPR effect might contribute to the huA33 accumulation and retention in the necrotic areas of the tumours, preclinical data suggest that this may be an antibody-specific effect. Ackerman et al. showed the ability of antibodies against GPA33 to progress towards a tumour spheroid centre even at very low concentrations [33]. They hypothesized that the slow rate of GPA33 turnover contributes to the ability to penetrate spheroids, allowing the bound antibody front to penetrate without being slowed by binding to replenish consumed antibody [33]. Aside from antigen turnover rate, it is also known that for an antibody of any given affinity, an increase in binding density is associated with significantly higher monoclonal antibody uptake in tumour [36-38]. This also appears to be true for huA33, with O’Donoghue et al. observing a linear relationship between ^124^I-huA33 uptake and antigen concentration in tumour, with estimated binding site occupancy for both tumour and normal colon of 20% to 50% [22]. Interestingly, O’Donoghue et al. obtained an antibody uptake of 0.1%ID/mL in focal regions with an average tumour uptake of 0.017%ID/mL which is among the highest reported for radiolabeled antibodies, as the authors concluded. HuA33 uptake observed in our study was similar to that reported by Welt et al. [39]. It is important to also note that unlike some antibodies against other tumour antigens [5], huA33 uptake appears to be universally and strongly present within human colorectal tumours, a result consistent with previous studies of murine A33 antibody *in vivo*[39]. Previous studies have demonstrated a high and uniform expression of GPA33 in colon cancers and throughout the normal intestinal mucosa [15,16]. Overall, it seems reasonable to hypothesize that the favourable GPA33 expression level and slow internalization rate are major contributors in determining ^131^I-huA33 uptake. This is highly relevant to the design of future studies investigating the therapeutic potential of huA33, particularly for payload delivery strategies. One obvious implication of our results is that patients with large or necrotic tumours should not be excluded from potential treatment with huA33 because of concerns of poor tumour cell targeting. Another is that combination therapy with antivascular agents will also require careful design given their potential effects on antibody penetration, as also shown in other studies [40,41] where bevacizumab treatment demonstrated to impair the penetration of antibodies into the tumour, resulting in a reduced tumour uptake of these antibodies.

The limit of our study is the small patient numbers, so further studies are needed to confirm the present findings.

## Conclusions

In summary, targeting GPA33 with huA33 presents a very attractive option given the universal and uniform expression of this antigen in colorectal cancers, and, within the limit of the study, the data presented here shows excellent uptake in primary and metastatic tumours of varying size and locations. Tumour cellular uptake is very high, even in the necrotic centres of tumours where tumour cell numbers and microvessel densities are very low, an unusual and therapeutically important finding for an intact IgG. These findings provide an important mechanistic support for ongoing clinical trials of huA33 in patients with metastatic colorectal carcinoma and provide data to rationally design them.

## Competing interests

The authors declare that they have no competing interests.

## Authors’ contributions

All authors have met the criteria for authorship. MC and GC collected, analyzed, and interpreted the data, and wrote the paper. HG interpreted the data and revised the manuscript critically. CM and FL analyzed the data and revised the manuscript. AC collected and analyzed data. DM collected and interpreted data. TJ collected and interpreted data. JH analyzed data and contributed to the manuscript writing. ME analyzed data and contributed to the manuscript writing. AB analyzed data and contributed to the manuscript writing. AS designed the study, interpreted the data, and revised the manuscript critically. All authors read and approved the final manuscript.
